# Ampelopsin Improves Insulin Resistance by Activating PPARγ and Subsequently Up-Regulating FGF21-AMPK Signaling Pathway

**DOI:** 10.1371/journal.pone.0159191

**Published:** 2016-07-08

**Authors:** Yong Zhou, Ying Wu, Yu Qin, Lei Liu, Jing Wan, Lingyun Zou, Qianyong Zhang, Jundong Zhu, Mantian Mi

**Affiliations:** 1 Research Center for Nutrition and Food Safety, Institute of Military Preventive Medicine, Third Military Medical University, Chongqing Key Laboratory of Nutrition and Food Safety, Research Center for Medical Nutrition, Chongqing, 400038, China; 2 College of Basic Medical Sciences, Third Military Medical University, Chongqing, 400038, China; Virgen Macarena University Hospital, School of Medicine, University of Seville, SPAIN

## Abstract

Ampelopsin (APL), a major bioactive constituent of *Ampelopsis grossedentata*, exerts a number of biological effects. Here, we explored the anti-diabetic activity of APL and elucidate the underlying mechanism of this action. In palmitate-induced insulin resistance of L6 myotubes, APL treatment markedly up- regulated phosphorylated insulin receptor substrate-1 and protein kinase B, along with a corresponding increase of glucose uptake capacity. APL treatment also increased expressions of fibroblast growth factor (FGF21) and phosphorylated adenosine 5’-monophosphate -activated protein kinase (p-AMPK), however inhibiting AMPK by Compound C or *AMPK* siRNA, or blockage of *FGF21 by FGF21* siRNA, obviously weakened APL -induced increases of FGF21 and p-AMPK as well as glucose uptake capacity in palmitate -pretreated L6 myotubes. Furthermore, APL could activate PPAR γ resulting in increases of glucose uptake capacity and expressions of FGF21 and p-AMPK in palmitate -pretreated L6 myotubes, whereas all those effects were obviously abolished by addition of GW9662, a specific inhibitor of peroxisome proliferator- activated receptor –γ (PPARγ), and *PPARγsiRNA*. Using molecular modeling and the luciferase reporter assays, we observed that APL could dock with the catalytic domain of PPARγ and dose-dependently up-regulate PPARγ activity. In summary, APL maybe a potential agonist of PPARγ and promotes insulin sensitization by activating PPARγ and subsequently regulating FGF21- AMPK signaling pathway. These results provide new insights into the protective health effects of APL, especially for the treatment of Type 2 diabetes mellitus.

## Introduction

Insulin resistance is regarded as hallmark for type II diabetes mellitus (T2DM), and plays an important role in the pathogenesis of the disease. Restoration of insulin sensitivity is a major strategy in the treatment of T2DM [1, 2]. The peroxisome proliferator- activated receptor-γ (PPARγ) is a clinically validated target for T2DM treatment and its agonists are clinically used to improve insulin resistance and counteract hyperglycemia [3, 4]. Currently available PPARγ agonists represented by thiazolidinediones (TDZs) (e.g. rosiglitazone, pioglitazone) are clinically effective insulin sensitizers [5–7].Whereas undesired side effects of TZDs (e.g. weight gain, cardiovascular risk, fluid retention) limit their clinical applications, thus, a need for a safer insulin sensitizer is apparent [5, 8–10]. *Ampelopsis grossedentata* is widely distributed in South China, and its tender stems and leaves are used as a healthy tea product named Rattan tea. Ampelopsin (APL), also named dihydromyricetin, one of flavonoids, is the major bioactive constituent of Ampelopsis grossedentata and exhibits outstanding anti-cancer, anti-inflammatory and anti-oxidative effects [11, 12]. Our previous experiments found that APL could significantly improved insulin resistance in rats with T2DM induced by low-dose streptozocin evidenced by decreasing the levels of blood glucose and serum insulin levels, serum insulin C-peptide and the homeostasis model assessment- insulin resistance (HOMA-IR). In this study, we would further verify the anti-diabetic activity of APL and elucidate the mechanism of this action. Interestingly, an increasing number of investigations have shown that naturally flavonoids (e.g. honokiol, kaempferol, galangin, quercentin, luteolin) were potent PPARγ agonists and have been known as attractive drug candidates for the therapy or prevention of T2DM with fewer unwanted side effects [13–17]. APL also belongs to flavonoids and its chemical structure was closely similar to quercentin and luteolin. For this reasons, we proposed that APL might also a prospective PPARγ agonist to regulate insulin sensitivity, glucose and lipid metabolism.

Fibroblast growth factor (FGF) 21, a novel member of the FGF family, has been identified as a potent metabolic regulator with pleiotropic effects on glucose and lipid metabolism. Initially, FGF21 is considered to be mainly synthesized and released by the liver and adipose tissues [18, 19]. But, recently, it has been found rodent skeletal muscle cells could be a source of FGF21, especially in response to insulin [20–22]. Reportedly, a large number of experiments have showed that FGF21 knockdown could increase PPARγ sumoylation which resulted in attenuating PPARγ-induced the beneficial insulin-sensitizing effects and increasing the detrimental side effects of the PPARγ agonist rosiglitazone, whereas adding back FGF21 could prevent sumoylation and restore PPARγ activity, therefore, FGF21 have been considered as a key mediator of the physiologic and pharmacologic actions of PPARγ [22–25].Moreover, numerous investigations have found that FGF21 regulates energy homeostasis through activation of AMP-activated protein kinase (AMPK) signaling pathway [26]. AMPK is a major metabolic energy sensor that regulates energy homeostasis and metabolic stress by controlling several homeostatic mechanisms that are acknowledged as other targets of T2DM treatment [27–29]. Our previous study has shown that APL supplementation could improve physical performance under acute hypoxic conditions partially by activation of AMPK in skeletal muscle [30].

Collectively, we hypothesized that APL maybe an approaching PPARγ agonist that beneficially improved insulin resistance. To clarify this hypothesis, the potential involvement of PPARγ activation and further modulation of FGF21-AMPK signaling pathway was evaluated in the models of skeletal muscle insulin resistance induced by palmitate. Our results indicated, for the first time, that APL maybe served as a PPARγ agonists and improved insulin resistance partially via activation of PPARγ and subsequent regulation of FGF21- AMPK signaling pathway.

## Results

### Ampelopsin improves palmitate -induced insulin resistance in skeletal muscle myotubes

**Fig 1:**
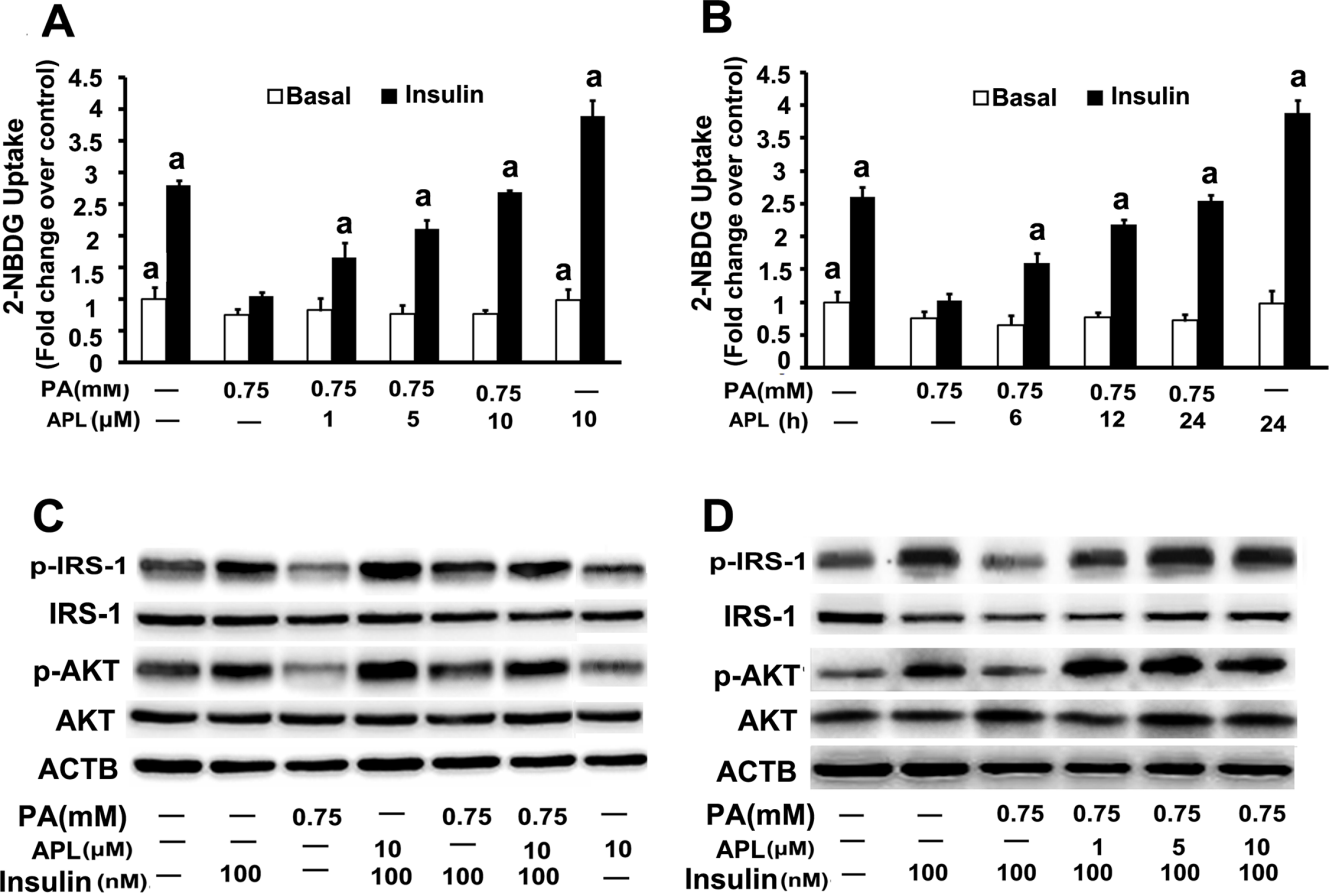
APL improved palmitate -induced insulin resistance in skeletal muscle myotubes. **(A)** Differentiated L6 cells were pretreated with palmitate (PA,0.75 mM) for 16 h, then incubated with 1, 5 or 10 μM APL for 24 h in the presence or absence of insulin (100 nM). Cells were collected and 2-NBDG glucose uptake was assessed. **(B)** Differentiated L6 cells were pretreated with palmitate (PA,0.75 mM) for 16 h, then incubated with APL (10 μM) for 6, 12 or 24 h in the presence or absence of insulin (100 nM). Cells were collected and 2-NBDG glucose uptake was assessed. **(C)** Differentiated L6 cells were untreated or pretreated with palmitate (PA,0.75 mM) for 16 h, then incubated with 10 μM APL for 24 h in the presence or absence of insulin (100 nM). IRS-1, p-IRS-1, Akt, and p-Akt were detected by western blot. **(D)** Cells were treated as described in (A). IRS-1, p-IRS-1, Akt, and p-Akt were detected by western blot. Values are means ± SEM. *n* = 3, ^a^*p* < 0.05 versus palmitate -treated group. A. U., arbitrary units. All results are representative western blots of three independent experiments with similar results.

Skeletal muscle insulin resistance is the primary defect in T2DM which has been considered to be an important target for T2DM prevention and treatment. For this reason, to confirm the contribution of APL to improve insulin resistance, glucose uptake capacity in palmitate -treated L6 myotubes was measured by 2-NBDG uptake. Differentiated cells were pre-incubated with palmitate (0.75 mM) for 16 h to induced insulin resistance as described before [31], then treated with different concentrations (1, 5 or 10 μM) of APL for 24 h or with 10 μM APL for different time intervals (6, 12 or 24 h) in the presence or absence of 100 nM insulin. We found that APL treatment had no significant effects on PA uptake outside the cells and had little impact on cell viability in L6 myotubes under the insulin-treated conditions and basal conditions (S1 and S2 Figs). Meanwhile, APL alone treatment could significantly increase glucose uptake capacity and expressions of p-IRS1 and p-Akt under insulin-treated conditions in L6 myotubes, but have no effects under the basal condition **(Fig 1A–1C)**.Under insulin- stimulated conditions, compared to the control group, palmitate treatment decreased the capability of glucose uptake in L6 myotubes, however APL treatment significantly increased the capability of glucose uptake in a time- and dose- dependent manner in palmitate -treated L6 myotubes **(Fig 1A and 1B).** We also measured the phosphorylated levels of IRS-1 (p-IRS-1) and Akt (p-Akt) proteins which are involved in insulin- signaling pathways. Expressions of p-IRS-1 and p-AKT were significantly inhibited by palmitate treatment and those effects were dose- dependently attenuated by APL treatment under insulin-stimulated conditions **(Fig 1C and 1D)**. These results suggested that APL could improve palmitate -induced insulin resistance in L6 skeletal muscle myotubes.

### Ampelopsin improves palmitate -induced insulin resistance via activating AMPK in skeletal muscle myotubes

**Fig 2:**
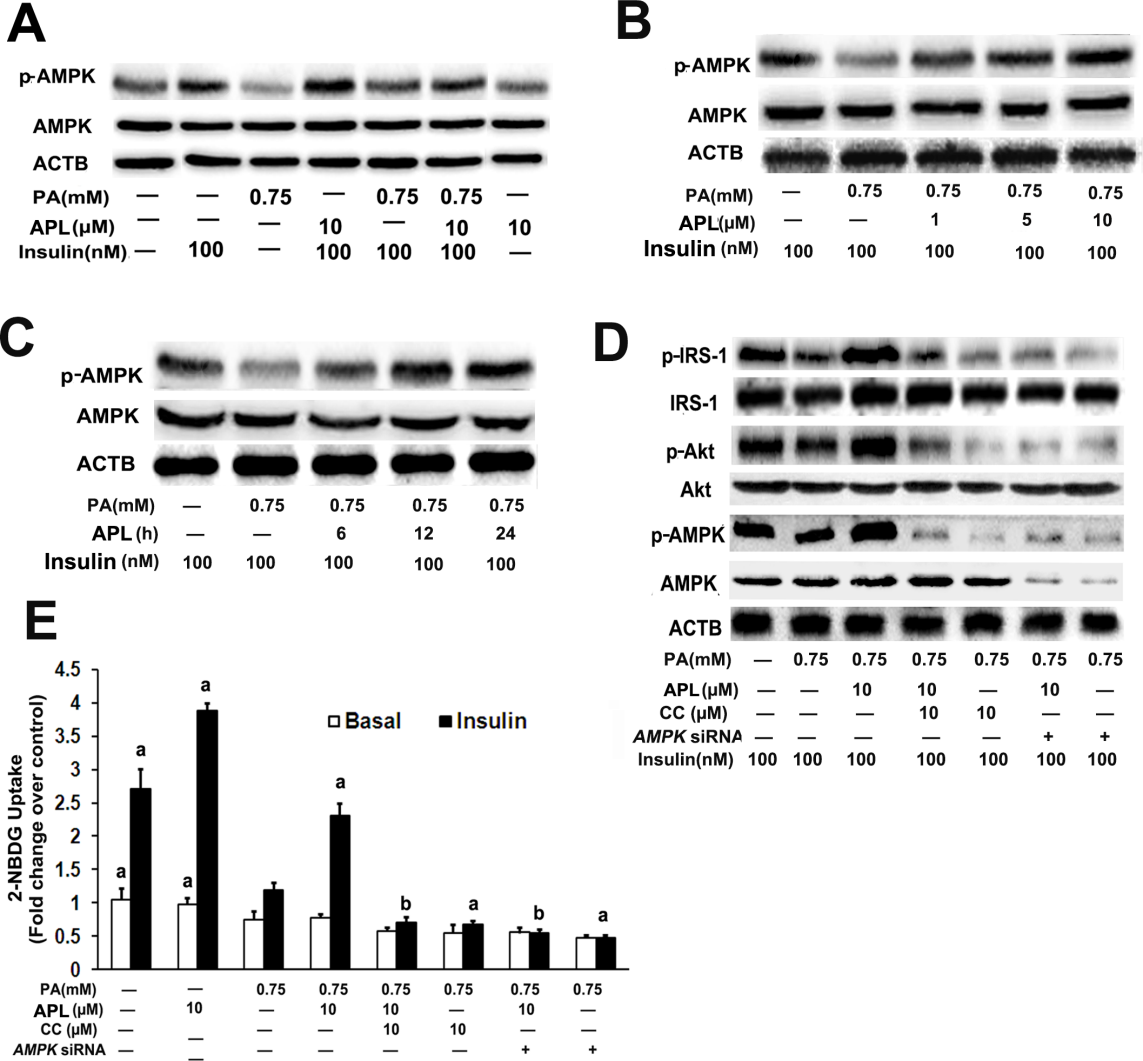
APL improved palmitate -induced insulin resistance through activating AMPK in skeletal muscle myotubes. **(A)**. Differentiated L6 cells were untreated or pretreated with palmitate (PA,0.75 mM) for 16 h, then incubated with 10 μM APL for 24 h in the presence or absence of insulin (100 nM). Western blots detected p-AMPK and AMPK **(B)** Differentiated L6 cells were pretreated with palmitate (PA,0.75 mM) for 16 h, then incubated with 1, 5 or 10 μM APL for another 24 h in the presence of insulin (100 nM). Western blots detected p-AMPK and AMPK. **(C)** Differentiated L6 cells were pretreated with palmitate (PA,0.75 mM) for 16 h, then incubated with (10 μM) of APL for 6, 12 or 24 h in the presence of insulin (100 nM). Western blots detected p-AMPK and AMPK. **(D)** Differentiated L6 cells were pretreated with palmitate (PA,0.75 mM) for 16 h, then with CC (10 μM) for 1 h or transfection with AMPK siRNA for 24 h,respectively, following by treated with 10 μM APL for 24 h in the presence or absence of insulin (100 nM). Total L6 cell lysates were used for western blots. **(E)** Differentiated L6 cells were treated as described in (D). Cells were collected and 2-NBDG glucose uptake was assessed. Values are means ± SEM. n = 3, ^a^*p* < 0.05 palmitate -treated group; ^b^*p* < 0.05 versus APL and palmitate co-treated group. All results are representative western blots of three independent experiments with similar results.

Given that AMPK play an important function in the regulation of energy homeostasis and metabolic stress, the role of AMPK in APL-mediated insulin sensitizing effects was investigated. Similarly, L6 myotubes were induced insulin resistance by palmitate as above mentioned, and APL treatment time- and dose-dependently increased p-AMPK expression in palmitate -treated L6 myotubes **(Fig 2A–2C)**. Then the AMPK-specific inhibitor was used for further investigation whether AMPK signaling pathway was involved in APL-mediated insulin sensitizing properties. As shown in Fig **2D and 2E**, blockage of AMPK by chemical inhibitors Compound C (CC) attenuated both APL-induced up-regulation of p-IRS-1 and p-AKT expression, and capability of glucose uptake in palmitate -treated L6 myotubes under insulin-stimulated conditions. As well, similar results were observed in shut down of AMPK by RNA interference. These results suggested that activation of AMPK signaling was required for APL-mediated insulin resistance improvement.

### FGF21 is involved in ampelopsin–induced AMPK activation

**Fig 3:**
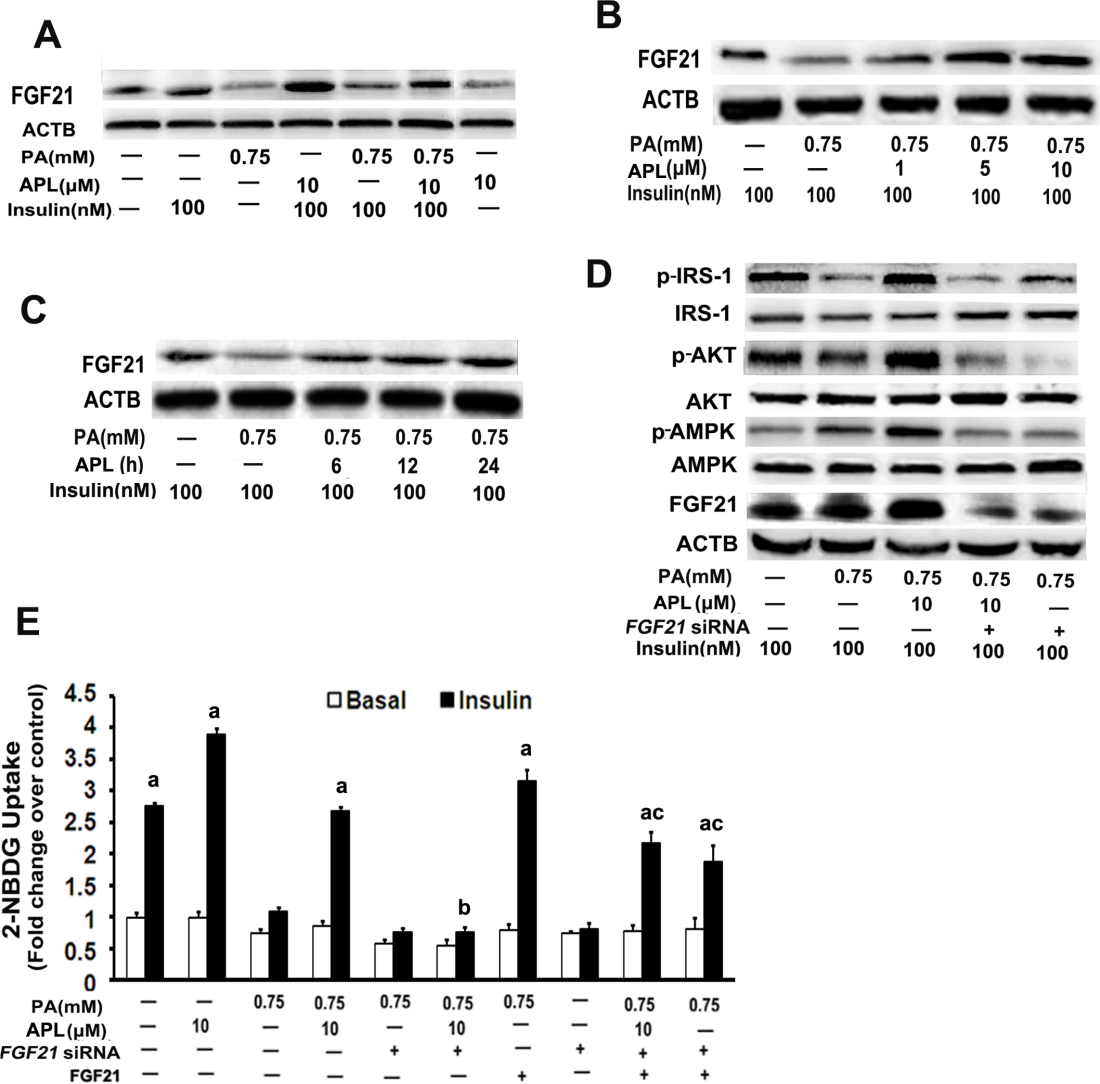
AMPK activation depended on APL-induced up-regulation of FGF21 expression in skeletal muscle myotubes. **(A)** Differentiated L6 cells were untreated or pretreated with palmitate (PA,0.75 mM) for 16 h, then incubated with 10 μM APL for 24 h in the presence or absence of insulin (100 nM). FGF21 expression was detected by western blot. **(B)** Differentiated L6 cells were pretreated with palmitate (PA,0.75 mM) for 16 h, and then incubated with 1, 5 or 10 μM APL for 24 h. FGF21 expression was detected by western blot. **(C)** Differentiated L6 cells were pretreated with palmitate (PA,0.75 mM) for 16 h, then incubated with (10 μM) of APL for 6, 12 or 24 h. FGF21 was detected by western blot. **(D)** Differentiated L6 cells were pretreated with palmitate (PA,0.75 mM) for 16 h, then transfected with *FGF21* siRNA before addition of APL (10 μM) for 24 h in the presence of insulin (100 nM). Total L6 cell lysates were used for western blots. **(E)** Differentiated L6 cells were pretreated with palmitate (PA,0.75 mM) for 16 h, then transfection with FGF21 siRNA for 24 h, following by treated with 10 μM APL, FGF21 protein (4.0μg/mL) or APL and FGF21 protein for 24 h, respectively,in the presence or absence of insulin (100 nM). Cells were collected and 2-NBDG glucose uptake was assessed. Values are means ± SEM. n = 3, ^a^*p* < 0.05 versus palmitate -treated group; ^*b*^*p* < 0.05 versus APL and palmitate co-treated group. ^c^*p* < 0.05 versus APL, *FGF21* siRNA and palmitate co-treated group. All results are representative western blots of three independent experiments with similar results.

FGF21 is a potent metabolic regulator with pleiotropic effects on glucose and lipid metabolism. It has been showed that FGF21 regulates energy homeostasis through activation of the AMPK signaling pathway. Therefore, we investigated whether FGF21 expression was implicated in APL-induced AMPK activation in palmitate -induced insulin resistance in skeletal muscle myotubes. As expected, APL treatment significantly enhanced FGF21 expression in a time- and dose-dependent manner in palmitate -treated L6 myotubes (**Fig 3A–3D**). Under insulin-stimulated conditions, *FGF21* siRNA transfection not only abolished APL-induced p-AMPK up-regulation, but also decreased APL-induced increase of glucose uptake and up-regulation of p-IRS-1 and p-AKT expression in palmitate -treated L6 myotubes (**Fig 3D and 3E**). Additionally, we found addition of FGF21 protein could rescues the reduction of 2-NBDG uptake by FGF21 knockdown in palmitate -treated L6 myotubes under insulin-stimulated conditions (**Fig 3E**). These data revealed that FGF21 was necessary for APL induced- AMPK activation and subsequent modulation of APL- mediated insulin sensitizing effects.

### Ampelopsin maybe a PPARγ agonist which is helpful for ampelopsin- mediated insulin sensitizing effects

**Fig 4:**
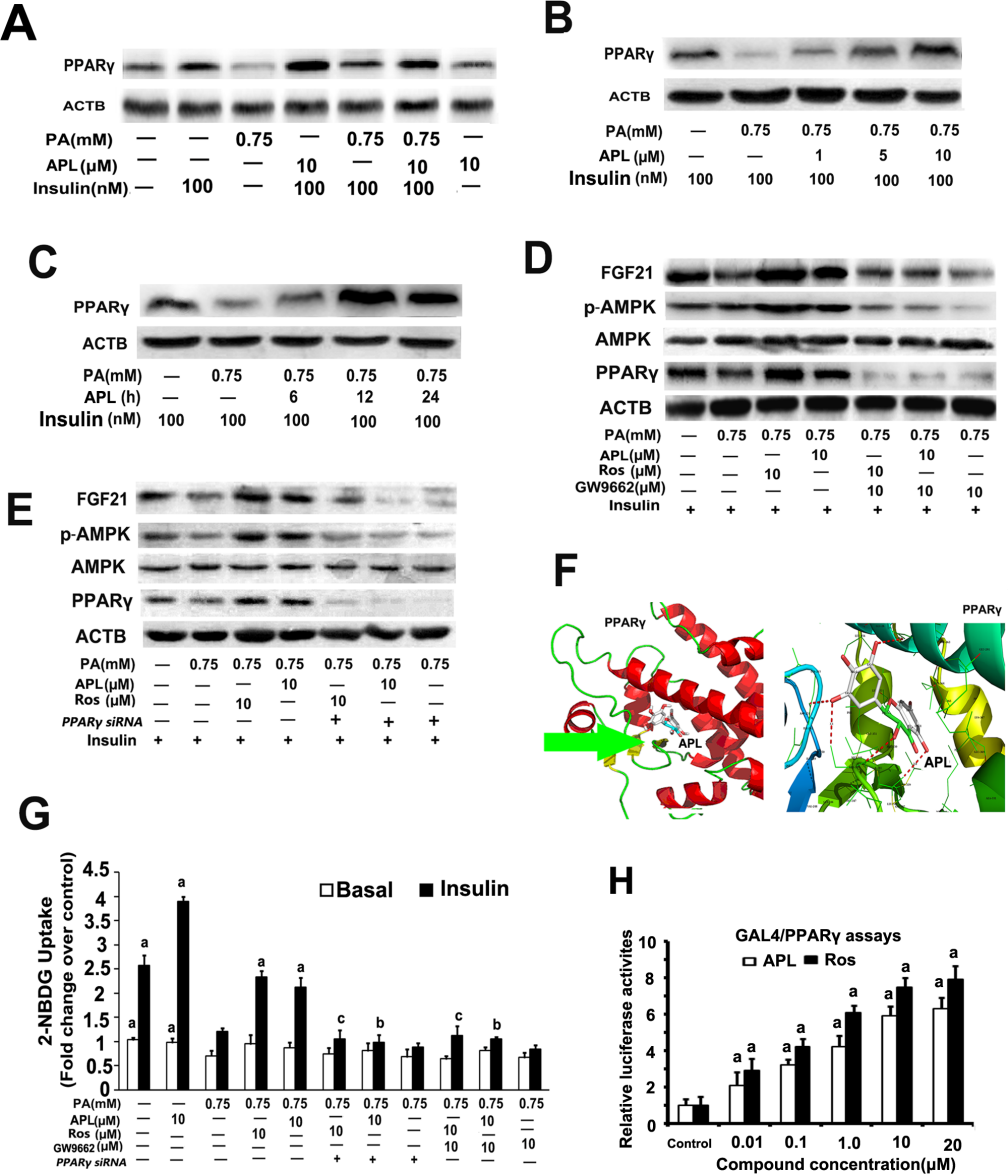
PPARγ activation was involved in APL-mediated FGF21 up-regulation in skeletal muscle myotubes. **(A)** Differentiated L6 cells were untreated or pretreated with palmitate (PA,0.75 mM) for 16 h, then incubated with 10 μM APL for 24 h in the presence or absence of insulin (100 nM). PPARγ was detected by western blot. **(B)** Differentiated L6 cells were pretreated with palmitate (PA,0.75 mM) for 16 h, and then incubated with 1, 5 or 10 μM APL for 24 h. PPARγ was detected by western blot. **(C)** Differentiated L6 cells were pretreated with palmitate (PA,0.75 mM) for 16 h, and then incubated with (10 μM) APL for 6, 12 or 24 h. PPARγ was detected by western blot. **(D)** Differentiated L6 cells were pretreated with palmitate (PA,0.75 mM) for 16 h, then cells were treated with GW9662 (10 μM) for 1 h, or following by treated with 10 μM APL or Rosiglitazone (Ros) (10 μM) for 24 h in the presence of insulin (100 nM). Total L6 cell lysates were used for western blots. **(E)** Differentiated L6 cells were pretreated with palmitate (PA,0.75 mM) for 16 h, then transfected with *PPARγ* siRNA for 24h, following by treated with 10 μM APL or Rosiglitazone (Ros) (10 μM) for 24 h in the presence of insulin (100 nM). Total L6 cell lysates were used for western blots. **(F)** Molecular modeling of the interaction between APL and PPARγ. A close-up view of the consensus orientation for APL is shown. The PPARγ protein is depicted as a ribbon representation and colored by secondary structures (i.e., helix, strand, and loop) (left panel); the hydrogen bonds between APL and PPARγ(distance<3.2 Å) are depicted as red dotted lines that include the names of the residues and distances (right panel). **(G)** Differentiated L6 cells were pretreated with palmitate (PA,0.75 mM) for 16 h, then cells were treated with GW9662 for 1 h or *PPARγ* siRNA for 24 h before addition of APL (10 μM) or Ros(10 μM) in the presence or absence of insulin (100 nM). Cells were collected and 2-NBDG glucose uptake was assessed. Values are means ± SEM. n = 3, ^a^*p* < 0.05 versus palmitate -treated group; ^b^*p* < 0.05 versus APL and palmitate co-treated group; ^c^*p* < 0.05 versus Ros and palmitate co-treated group. All results are representative western blots of three independent experiments with similar results. (H)Activation effect of APL and rosiglitazone (Ros) on hPPARγ. The activity of the vehicle control was set at 1 and the relative luciferase activities are presented as fold induction relative to the vehicle control. ^a^*p* < 0.05 versus control group. n = 3. Mean±SD.

PPARγ ligands have emerged as potent insulin sensitizers which have been used as highly effective oral medications for T2DM. Recently, naturally occurring flavonoids(e.g. quercentin, luteolin) with similar chemical structure to APL have been found to be potent PPARγ partial agonists for modulation of lipid and glucose metabolism [13, 14]. Consequently, we investigated whether PPARγ activation is related in APL-induced insulin resistance improvement in palmitate -treated L6 myotubes. As expected, APL treatment up-regulated PPARγ expression in a time-dependent and dose-dependent manner in palmitate -treated L6 myotubes **(Fig 4A–4C)**. Blocking PPARγ by its special inhibitor GW9662 or *PPARγ siRNA* significantly not only abolished APL-induced FGF21 up-regulation, but also inhibited APL-induced AMPK activation **(Fig 4D and 4E)**. Meanwhile, GW9662 or *PPARγ siRNA* treatment significantly attenuated APL- induced insulin resistance improvement as evidenced by decrease of glucose uptake capability in palmitate -treated L6 myotubes **(Fig 4G)**.

To further elucidate whether APL could bind directly to the active site of PPARγ, similar to quercentin and luteolin, molecular docking methods were used to study the interaction between APL and PPARγ. Free-binding energy was calculated five times, and the lowest binding energy (-7.7 kcal/mol) was used as the affinity score for APL and PPARγ interactions. APL docked into the catalytic site of PPARγ, suggesting that APL was a PPARγ agonist (**Fig 4F**). Furthermore, we used the luciferase reporter assays to determine if APL has PPARγ agonist activity. We found, similar to PPARγ agonist rosiglitazone, APL was a potent activator of PPARγ and dose-dependently upregulated luciferase activity (**Fig 4H**).

Collectively, these findings above indicated that APL- mediated insulin resistance improvement maybe, at least in part, be attributed to the activation of PPARγ.

## Discussion

Here, for the first time, we provide evidence that APL could improve insulin resistance, partially through activating PPARγ and subsequently regulating FGF21-AMPK signaling pathway. This novel finding is approved by the following evidences: 1) APL treatment notably time- and dose-dependently enhanced glucose uptake capability and up-regulated of p-IRS-1 and p-Akt proteins in palmitate -induced insulin resistance of L6 skeletal muscle myotubes; 2) AMPK activation resulted in APL-induced insulin resistance improvement; 3) FGF21 was involved in APL–induced AMPK activation; and 4) APL was a potential PPARγ agonist, and PPARγ activation was required for APL-induced FGF21- AMPK signaling pathway.

Improving insulin resistance is the most important strategy for the prevention and treatment of T2DM. Naturally occurring plant compounds particularly flavonoids are attractive candidates because of their abundance in nature, inexpensiveness to produce and fewer side effects than currently used pharmaceutical agents in clinical therapy[14, 17]. Ampelopsin (APL) belongs to the natural flavonids and is the major bioactive component extracted from Chinese medicinal herb Ampelopsis grossedentata, which is widely grown in South China and its tender leaves and stems are used as a healthy tea product named Rattan tea. Reportedly, APL has diverse healthy benefits including anti-oxidative, anti-cancer and hepato-protective activities [11, 12, 32]. Here, our study found that APL could improve insulin resistance which was approved by the following evidences: 1) APL treatment had notably beneficial effects on enhance glucose uptake capability in the models of skeletal muscle insulin resistance induced by palmitate; 2) APL treatment up-regulated of p-IRS-1 and p-Akt proteins which are involved in insulin- signaling pathways. These results suggested that APL might be a potent therapeutic agent for T2DM prevention and treatment.

We investigated the underlying mechanisms of APL-induced insulin resistance improvement. It is well known that AMPK is a key regulator for energy metabolism and a therapeutic target for T2DM because of its anti-insulin resistance properties. Recently, many flavonoids such as kaempferol and galangin were found to activate AMPK [33, 34]. Our previous study has found that APL supplementation could improve physical performance under simulated high-altitude conditions, partially through activation of AMPK in skeletal muscle [30]. Here, we also report that APL could activate AMPK in palmitate -treated skeletal muscle myotubes, and blockage of AMPK by AMPK inhibitor CC or AMPK siRNA significantly abolished the effects of APL on insulin resistance improvement, indicating that AMPK was involved in APL-induced insulin resistance improvements.

In addition to AMPK, our results supported an important role for a PPARγ-FGF21 associated pathway in APL mediated- insulin resistance improvement. PPARγ ligands have shown great promise for therapeutic interventions in metabolic disorders such as T2DM. However, in spite of being effective in normalization of blood glucose levels, so far experienced unwanted side effects of the currently used PPARγ agonists from TDZs, promote the search for new PPARγactivators [5, 9, 35]. Reportedly it was found that PPARγ could induce FGF21 which in turn amplified PPARγ activity and promoted insulin sensitization, whereas blockage of FGF21 could lead to decrease the insulin- sensitizing effects of TDZs as well as increasing the associated weight gain and fluid retention, implicating FGF21 as a vital mediator of the anti-diabetic actions and negative side effects of TDZs [5, 23–25]. In the past few years, many new natural candidates of PPARγ lingand have been confirmed such as quercentin and luteolin which can modulate lipid and glucose metabolism with few side effects of TDZs [14–17]. In our study, APL treatments significantly enhanced FGF21 expression, but blockage of FGF21 by FGF21 siRNA decreased APL-induced up-regulation of FGF21 and p-AMPK, along with a decrease in glucose uptake capability of palmitate -treated L6 myotubes. Moreover, APL treatments also activated PPARγ, whereas pretreated with GW9662, a specific inhibitor of PPARγ, or blocking PPARγ using RNA interference could notably inhibit APL-induced PPARγactivation which resulted in a consequence of weakening APL-induced up-regulation of FGF21 and p-AMPK expressions and decreasing APL-induced a increase in glucose uptake capacity of palmitate -treated L6 myotubes. Furthermore, using molecular modeling, as expected, APL, similar to luteolin which has been confirmed as PPARγ ligand[15, 17], directly bound to the PPARγ catalytic site. Furthermore, the luciferase reporter assays have found APLcould activate luciferase activity in a dose- dependent manner. All these results suggested that APL might be a PPARγ agonist and PPARγ-FGF21 might be a major signaling pathway that mediates APL-induced AMPK activation and insulin sensitivity in palmitate in skeletal muscle myotubes.

In conclusion, this study suggested that APL maybe a potential PPARγ agonist to improve insulin resistance via activating PPARγ and further regulating FGF21-AMPK signaling pathway, which therefore provide experimental evidences for developing APL as an attractive therapeutic drug for prevention and treatment of T2DM and other insulin resistance-related metabolic diseases such as nonalcoholic fatty liver disease.

## Materials and Methods

### Antibodies and reagents

Compound C (CC; P5499), Palmitate (P5585), FGF21 protein (SRP6184), Minimum Essential Medium Eagle (MEM; M4526),Rosiglitazone(Ros, R2408) were purchased from Sigma- Aldrich. GW9662 (70785) was got from Cayman chemical Company (Ann Arbor, MI). 2-[N-(7-Nitrobenz- 2-oxa-1,3- diazol-4-yl)amino]-2-deoxyglucose (2-NBDG; N13195) and Lipofectamine™ 2000 transfection reagent (11668–019) were purchased from Invitrogen. Fetal bovine serum (FBS; SH30370.03) was purchased from Hyclone Laboratories. Antibodies against p-AMPK (sc-33524), AMPK (sc-74461), insulin receptor substrate-1 (IRS-1; sc-599), p-IRS-1 tyr465 (sc-3956), siRNAs for *PPARγ(sc-156077)*,*FGF21 (sc-156171)* and *AMPK (sc- 155985)*, control (sc-44230) were obtained from Santa Cruz Biotechnology. Antibodies against protein kinase B (Akt; 9272) and p-Akt (9271) were obtained from Cell Signaling Technology. Antibodies against ACTB/β-actin (TA-09) was obtained from Zhongshan Jinqiao Biotechnology Co. APL (msat-120131108, HPLC≥98%) was purchased from Chengdu MUST Bio- Technology co., LTD.

### Cell culture and treatments

Rat skeletal muscle L6 myoblast cells were purchased from Institute of Biochemistry and Cell Biology, Chinese Academy of Sciences (Shanghai, China), and were maintained in MEM containing 10% FBS and 1% antibiotic/antimycotic solution (10,000 U/mL penicillin and 10 mg/mL streptomycin) at 37°C in a humidified atmosphere with 5% CO_2_. Cells were differentiated to myotubes staged in medium supplemented with 2% FBS as decribed before [36]. Then, the differentiated myotubes were used for the next exprements.

### Palmitate preparation

PA was dissolved in 40 mL of 0.1 M NaOH at 70°C. BSA solution (1%) was prepared in distilled water. PA (0.75 mM) was used for treatment of L6 myotubes after conjugation with 1% BSA on a magnetic stirrer set at 40°C.

### 2-NBDG glucose uptake assay

L6 myotubes were exposed to the indicated treatments for 24 h and 100 mM 2-NBDG was added to the media for 2 h, followed by washing with PBS three times. The collected myotubes were analyzed using a laser confocal microscope and flow cytometry detection analyzer.

### RNA interference

Control non-targeted siRNA or siRNA against *PPARγ*, *FGF21*, *AMPK* and Lipofectamine 2000 were diluted in reduced serum MEM according to the manufacturer’s protocol. The final siRNA concentration was 100 nM and plasmid concentration was 4 μg. L6 myotubes were incubated with the transfection mixture for 10 h and then supplemented with fresh medium for an additional 24 h, and exposed to the indicated treatments. Thereafter, cells were harvested for western blots.

### Western blotting

Total cell lysate was analyzed by western blot analysis as previously described [11, 12]. Briefly, the protein samples extracted from L6 myotubes were subjected to sodium dodecyl sulfate polyacrylamide gel electrophoresis and transferred to a polyvinylidene difluoride membrane. Blots were probed with 1:1, 000-diluted primary antibodies overnight at 4°C, followed by horseradish peroxidase- conjugated secondary antibodies (Thermo Scientific Lab Vision; 31340 and 31455). Visualization used an enhanced chemiluminescence system (VILBER Fusion FX7, France). Densitometric analysis was performed using Scion Image-Release Beta 4.02 software (http://scion-corporation.informer.com/).

### In Silico Molecular Modeling Studies of PPARγ

Based on the ‘lock-and-key’ principle of interaction between ligands and receptors, molecular docking method, which simulate the interaction between a small molecule ligand and a bio-macromolecule receptor, was used to investigate the interaction between APL and PPARγ as described [37]. Briefly, we determined the 3D structure of APL based on initial molecular data from PubChem (PubChem ID 161557). A structural model of the catalytic domain of PPARγ was constructed using Auto Dock Tools from the published crystal structure of PPARγ (PDB ID 1ZGY) as the modeling template. NAMD (version 2.7) was employed during the molecular dynamics simulation to obtain a refined structure. During the molecular dynamics simulations, the entire structure was surrounded by a cubic water box of simple point charge (SPC) water molecules that extended 10 Å from the protein, and periodic boundary conditions were applied in all directions. The systems were neutralized with Na^+^ and Cl^-^ counter ions that replaced the water molecules. Energy minimization was performed for 5000 steps, followed by a 500-ps production molecular dynamics simulation with a time-step of 2 fs at constant pressure (1 atm) and temperature (300 K). Furthermore, docking parameters were adjusted to enable the search space of Autodock-Vina to include the potential binding region of APL. In our docking computation, we assumed that APL would interact with PPARγ via the catalytic domain. The binding energy of PPARγ and APL was calculated using Autodock-Vina software assuming the lower the binding energy, the higher the affinity of a particular combination [38].

### Luciferase reporter assay

The pM-hPPARγ(a chimera protein expression plasmid for the GAL4 DNA-binding domain and human PPARγ ligand-binding domain), pUAS(5x)-tk-luc (a reporter plasmid) and pRL-CMV-Rluc(an internal control plasmid for normalizing transfection efficiency) were transfected into HEK-293 cells by using the Lipofectamine 2000 system (Invitrogen, USA) overnight and then removed. APL and rosiglitazone were diluted in fresh media, and then added into the cells. After incubating for another 24 h, the cells were lysed and analyzed by using a dual-luciferase reporter gene assay system (Promega, USA) according to the manufacturer’s protocol. All the transfection experiments were repeated at least three times independently in triplicate.

### Statistical analysis

Quantitative data are presented as means ± standard deviation (SD) of three experiments. Statistical analyses were performed by *t*-test and one-way analysis of variance using SPSS 13.0 statistical software (SPSS Inc., Chicago, IL, USA). A *p*-value <0.05 was considered statistically significant and the Tukey-Kramer post-hoc test was applied if *p* < 0.05.

## Supporting Information

S1 Fig Effect of APL on palmitate uptake outside the cells. (TIF)

S2 Fig Effect of APL on cell viability in L6 myotubes. (TIF)

## Abbreviations

APL: Ampelopsin
AMPK: 2-NBDG,2-deoxy-2-[(7-nitro-2,1,3-benzoxadiazol-4-yl)amino]- D- glucose
AMP: activated protein kinase
Akt: Protein kinase B
CC: compound C
FGF21: Fibroblast growth factor
IR: Insulin receptor
PPAR: Peroxisome proliferator- activated receptor
siRNA: small interfering RNA
T2DM: Type 2 diabetes mellitus
FPG: fasting plasma glucose
HOMA-IR: homeostasis model assessment of insulin resistance
