# Supplementary figures and images for: Ampelopsin Improves Insulin Resistance by Activating PPARγ and Subsequently Up-Regulating FGF21-AMPK Signaling Pathway
Source: PLoS One. 2016 Jul 8;11(7):e0159191. doi: 10.1371/journal.pone.0159191 (PMC4938387; doi:10.1371/journal.pone.0159191)

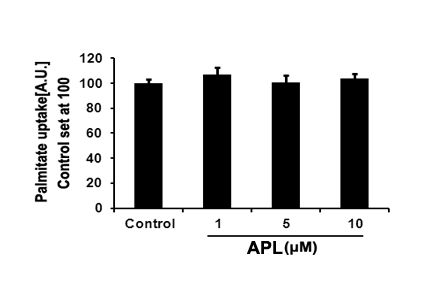

Supplement: S1 Fig — (TIF) [file pone.0159191.s001.tif]

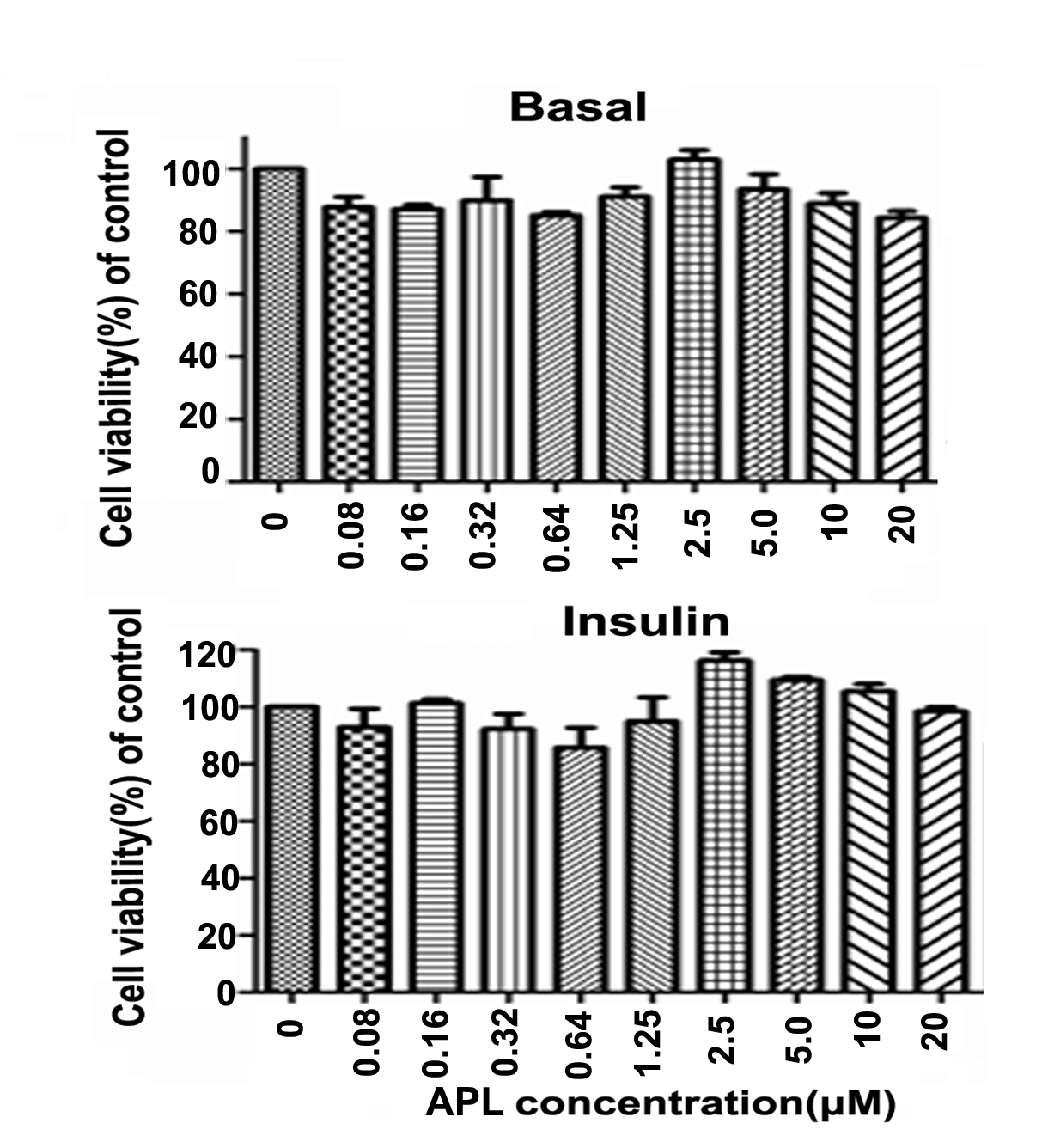

Supplement: S2 Fig — (TIF) [file pone.0159191.s002.tif]
